# Change of antidepressant utilization in children, adolescents and young adults in Europe before and during the COVID-19 pandemic: a systematic review

**DOI:** 10.1007/s00787-025-02839-x

**Published:** 2025-08-14

**Authors:** Alexander M. Fassmer, Kathrin Wandscher, Aida Bedri, Kathrin Jobski, Luise Poustka, Christian J. Bachmann, Falk Hoffmann

**Affiliations:** 1 Department of Health Services Research, Division of Outpatient Care and Pharmacoepidemiology, Carl von Ossietzky Universität Oldenburg, Oldenburg, Lower Saxony Germany; 2 Department of Health Services Research, Division of General Practice/Family Medicine, Carl von Ossietzky Universität Oldenburg, Oldenburg, Lower Saxony Germany; 3 Clinic for Child and Adolescent Psychiatry, Heidelberg University Hospital, Heidelberg, Baden-Wuerttemberg Germany; 4 Department of Child and Adolescent Psychiatry, University Hospital Ulm, Ulm, Baden-Wuerttemberg Germany

**Keywords:** Antidepressants, Children, Adolescents, Young adults, Europe, COVID-19 pandemic

## Abstract

**Background:**

In recent decades, antidepressant utilization among young persons in Western countries has increased, raising concerns about overprescribing and safety. The COVID-19 pandemic and respective restrictions might have impacted not only youth’s mental health but also antidepressant prescribing. Our aim was to systematically investigate changes in antidepressant utilization during the pandemic compared to pre-pandemic periods in European young persons.

**Methods:**

This systematic review was registered in PROSPERO (CRD42024559951). Observational studies with ≥ 100 European young persons (0–24 years) reporting prevalence or incidence data in antidepressant utilization before and during the pandemic (2018/2019 vs. 2021/2022) were included and percentage changes between two time periods calculated. MEDLINE (via PubMed), PsycINFO, and EMBASE were searched from January 1, 2021 to July 3, 2024 and supplemented by citation searching. Study quality was assessed using the Joanna Briggs Institute’s tool.

**Findings:**

We screened 4,416 records for eligibility and included eight studies covering data from Austria, Denmark, Finland, France, Italy, Norway, Spain, and Sweden (*n* = 4 from Nordic countries). The number of included young persons ranged from 1071 to 3,455,521 and all studies used secondary data, mostly from registries. All studies showed a relative increase in overall antidepressant use during the COVID-19 pandemic, with variability between countries ranging from 23 to 52%. Antidepressant utilization showed higher increases in adolescents (*n* = 3 studies) and females (*n* = 3 studies). Selective serotonin reuptake inhibitors were more common (73.9–90.9%; *n* = 3 studies) than other antidepressant classes.

**Interpretation:**

During the COVID-19 pandemic, antidepressant utilization in young persons increased modestly in all studied European countries. This increase may mirror the surge in mental health problems in young persons during the pandemic, but may also reflect altered patterns of mental health services availability.

**Supplementary Information:**

The online version contains supplementary material available at 10.1007/s00787-025-02839-x.

## Introduction

The use of antidepressants in children, adolescents and young adults (young persons) is a much-discussed topic that continues to attract attention from both experts and the public due to concerns about efficacy, safety, and long-term impacts [1, 2]. Antidepressants are commonly prescribed to treat depression, anxiety and other affective disorders that are prevalent among young persons [3, 4]. In recent decades, the use of antidepressant drugs in young persons has increased steadily in most Western countries [5–7]. In Europe, national data has documented a consistent rise in antidepressant use among young persons throughout the 2010s. In Sweden, the prevalence of antidepressant use among 0–24-year-olds increased from 1.4 to 2.1% between 2006 and 2013 [8]. In the United Kingdom, the rate of new antidepressant prescriptions for 12- to 17-year-olds more than doubled between 2005 and 2017, reaching 9.7 prescriptions per 1,000 persons for females and 4.2 for males [9].

While these secular trends in antidepressant prescribing were already underway, the COVID-19 pandemic introduced a disruptive event that may have further influenced mental health treatment patterns. Although the United Nations (UN) World Health Organization (WHO) declared the end of the global COVID-19 pandemic in May 2023, marking the end of the public health emergency [10], the pandemic has placed an unprecedented and continuing burden on people of all ages. Young persons were particularly affected by the restrictions on their social lives, school, university and business closures and the loss of familiar leisure activities [11] and this extraordinary situation affected mental health considerably [12, 13]. Following the onset of the pandemic, increased prescribing of antidepressants in the general population [14, 15] and in young persons [13, 16] have been reported in several studies from different European countries with various findings. This was, for instance, demonstrated by Tiger et al. [17], who analyzed antidepressant use in Denmark, Sweden and Norway across all age groups, including children and adolescents, and found a marked increase during the initial pandemic year (2020) compared to 2015–2019. The data revealed that Sweden exhibited the most striking relative increase in use (+ 33%) in young persons aged 0–19 years. Additionally, Sweden demonstrated a higher overall use than the other two countries. Although the number of studies carried out in Europe is constantly increasing, the studies differ in the selection of the population, the measurement of antidepressant use and the quality. Moreover, a comprehensive overview for assessing the changes of use among European countries is still lacking.

Therefore, this systematic review aims to identify and synthesise observational evidence on changes in antidepressant utilization among European young persons during the COVID-19 pandemic compared to pre-pandemic periods.

## Methods

This systematic review was conducted in accordance with the methodological framework outlined by the Joanna Briggs Institute (JBI) for systematic reviews of prevalence and incidence [18]. We report this review according to the Preferred Reporting Items for Systematic Reviews and Meta-Analyses (PRISMA) statement [19] and its protocol has been registered in PROSPERO (CRD42024559951).

### Data sources and search

We searched the following electronic bibliographic databases starting with the publication date January 1, 2021 to July 3, 2024: MEDLINE (via PubMed), PsycINFO (via EBSCOhost) and EMBASE (via Elsevier). Search strategies used in other systematic reviews for antidepressants [20, 21], drug prescriptions [22] and children/adolescents/young adults [23, 24] were adapted. Full details of the search strategies can be found in supplementary file S1. We did not include terms such as ‘COVID-19’, ‘SARS-CoV-2’ or ‘pandemic’ in our search syntax, instead applying a broader search. Our aim was to identify all studies analyzing antidepressant utilization over time, regardless of whether the change was explicitly attributed to the pandemic. To supplement the database searches, we performed a forward (citing) and backwards (cited) citation analysis of the included studies using Web of Science Core Collection on September 23, 2024. This was repeated iteratively on newly identified eligible references until no further could be identified.

### Eligibility criteria

We defined the pre-pandemic period as the calendar years 2018 and 2019, representing a stable baseline before major public health interventions were implemented. Although the COVID-19 pandemic began in late 2019 and accelerated in early 2020, we excluded 2020 as a transitional year due to high heterogeneity in the timing and intensity of restrictions across countries. The pandemic period was defined as 2021 and 2022, when the full effects of the pandemic and associated containment measures were ongoing. If information was available for multiple time intervals in a period (e.g., for 2018 and 2019 as well as for 2021 and 2022), the most recent information before the pandemic was compared to the least recent during the pandemic (i.e. 2019 to 2021). We included published observational studies with a sample size of ≥ 100 participants at each of the 2 required time points to ensure a minimum level of statistical stability and population representativeness. Interventional studies, PhD theses, and conference abstracts were excluded. There were no language restrictions.

We used the CoCoPop (condition, context, population) mnemonic recommended for reviews assessing prevalence and incidence data to set inclusion and exclusion criteria [25].

### Condition

Studies investigating the change in antidepressant utilization (at least one agent) before and during the COVID-19 pandemic were included if they reported point prevalence, period prevalence or incidence. The numerator could contain the number of users or another measure of usage, the denominator had to contain either number of observed persons or person time. Studies that analyzed trends without reporting these metrics, or that only attempted to predict trends, were excluded. The same measure and the same time interval (e.g., one year or one month) had to be available for both periods (e.g., one-year prevalence in 2019 compared to 2022).

### Context

We included population-based studies, including school-based or community samples in Europe. European countries were considered based on the UN geoscheme devised by the UN Statistics Division [26]. The restriction to Europe was chosen because many European countries employed similar responses to COVID-19 [27]. Their approach to the pandemic was different from other parts of the world, such as China [28]. Besides, there were major differences in comparison with the USA, where the excess all-cause mortality rate was considerably higher than in Europe [29].

### Population

For inclusion, studies had to report data on young persons between 0 and 24 years as defined by the UN [30]. This age range was chosen to reflect the broad impact of the pandemic across developmental stages and to capture the full spectrum of antidepressant utilization patterns in young persons. Studies reporting on only part of this age group (e.g., 12–18 years) were also included. Studies were excluded if they reported a broader age range (e.g., 20 years and older) but did not explicitly report on our target age group (in this example, 20–24 years). The studies to be included should have examined general populations. Studies limited to specific groups of young persons like those with specific diseases (e.g., depression or anxiety disorder) or those in specific care settings (e.g. hospital) were excluded.

### Data selection and extraction

The search results were imported into an EndNote library (version 20.5, Clarivate, Philadelphia, PA, USA) and then into Rayyan [31] after removal of duplicates. The screening process was then piloted on 100 titles/abstracts by AF, AB, KJ and FH. Thereafter, AF and one other reviewer (AB, KJ, or FH) screened the remaining title/abstracts. The full text of all articles that met the inclusion criteria were also assessed independently by AF and AB. Any disagreement was resolved by discussion or by a third reviewer (KJ or FH).

The following data were extracted: authors, year of publication, country, data source, year(s) of data collection, number of included persons or person-time, inclusion criteria, characteristics of included persons (age, sex), data on utilization of antidepressants before and during the pandemic. If studies with samples from databases or registries did not report the number of included persons, external sources (i.e., statistical database or registries from the respective country) were sought and extracted to provide an approximate number of the study population. If studies only presented findings in figures on utilization of antidepressants, we contacted the study authors for this information once. If study authors did not respond, we extracted the data from the figures independently by two reviewers (AF and AB) using WebPlotDigitizer by automeris (https://automeris.io/). This process was previously piloted on two articles. One author (AF) extracted data and entered it into the results tables shown below. This process was piloted on two articles before. The extracted data were verified by a second reviewer (AB). Any disagreement was resolved by discussion or by a third reviewer (KJ).

### Quality assessment

Two reviewers (AF and KW) independently assessed the quality of the included studies using the JBI’s critical appraisal checklist for studies reporting prevalence data [25], which was adapted for our research question (supplementary file S2). The checklist includes nine items with the response options “Yes” and “No” (items 1–9) as well as “Unclear” (items 1–3, 5–7, and 9) and “Not applicable” (items 2 and 9). The assessment was piloted to calibrate the reviewers using two of the included studies. Any disagreement was resolved by discussion.

### Data synthesis

A formal narrative synthesis was conducted. A meta-analysis was not planned because of the expected methodological heterogeneity in measuring changes in antidepressant use between studies. The baseline characteristics and findings of the included studies were summarized descriptively. Across included studies, antidepressant utilization was reported for various age subgroups. Where data were available, we extracted and reported results stratified by age (e.g., 6–11 years, 12–17 years, 18–24 years) to reflect heterogeneity within the 0–24 age span. In order to demonstrate the change between the two utilization measures prior to and during the pandemic, we calculated the relative change in percent. This was done by subtracting the pre-pandemic measure from the during-pandemic measure and dividing the result by the pre-pandemic measure. Given the considerable diversity of the populations in Europe, the country was used for the purpose of grouping the included studies for the narrative synthesis.

## Results

### Study selection

**Fig. 1:**
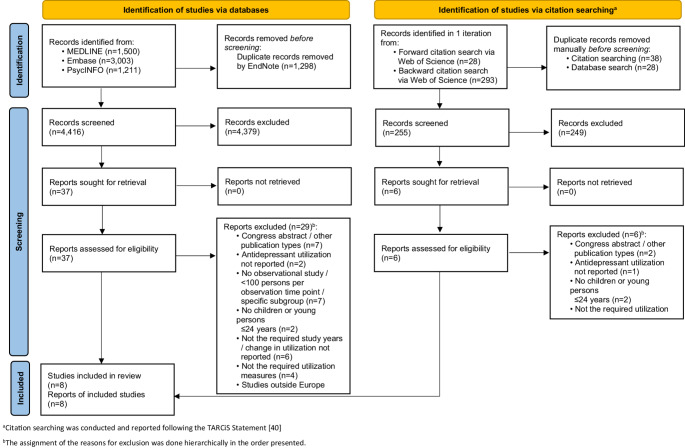
Flow diagram

The database search identified 4,416 records (see Fig. 1). Following title/abstract screening, 37 full texts were reviewed, and eight articles reporting on eight studies were included [32–39]. No additional articles were identified through the citation searching process (one iteration). All eight articles were written in English. Supplementary file S3 shows all records which were excluded after the full text screening, with reasons for exclusion.

### Study and participant characteristics

The characteristics of all included studies are summarized in Table 1. Among these, six examined data from a single country [32, 34–37, 39], while two studies encompassed data from multiple countries [33, 38]. The countries for which data were analyzed were Austria, Denmark, Finland, France, Italy, Norway, Spain and Sweden.

**Table 1:** Baseline characteristics of the data included

First author, year	Country/ Countries included in review	Data source and study design	Period of data collection	Inclusion criteria	Antidepressant utilization	Used measure	Sample size with time reference (number of included children/adolescents/ young adults)	Mean/median age, years; proportion of females
Armando, 2024 [32]	Italy	- Electronic health records of the ASL T04 (local health authority for the districts of Ciriè, Chivasso, Settimo Torinese, San Mauro, Ivrea and Cuorgnè) in the Piemonte region - Retrospective cohort study	Jan 2018 – Nov 2021	Young adults (18–22 years)	≥ 1 dispensation of an antidepressant within ATC N06A including N06AA (NSMRIs), N06AB (SSRIs), N06AX (other antidepressants)	Weekly prevalence	1,071 young adults (time reference n.r.)	n.r.; 60.3%
Bojanić, 2024 [33]	Denmark, Sweden	- National prescription registers of Denmark (The Danish Register of Medicinal Product Statistics), Sweden (The Swedish National Prescribed Drug Register) and the Nordic Medico-Statistical Committees database* - Repeated cross-sectional study	2006–2021	Individuals (age groups 5–19, 20–64, ≥ 65 years)	≥ 1 dispensation for an antidepressant within ATC N06A including N06AA (NSMRIs), N06AB (SSRIs), N06AX (other antidepressants)	1-year prevalence	n.r., but two external sources identified [41, 42]: Denmark: approx. 980,400 5-19-year-olds (on January 1, 2021) Sweden: 1,841,640 5-19-year-olds (on January 1, 2021)	n.r.
Garcia, 2023 [34]	Spain	- All dispensed medications for the primary care setting in the Asturias region - Repeated cross-sectional study	2018–2021	Individuals (age groups 0–14, 15–29, 30–44, 45–59, 60–74, 75–89, ≥ 90 years)	≥ 1 dispensation of an antidepressant within ATC N06A	Defined Daily Doses × 1,000 inhabitants∕total population × time (days)	n.r., but one external source identified [43]: approx. 105,266 0-14-year-olds (on July 1, 2021)	n.r.
Kuitunen, 2022 [35]	Finland	- Statistics on reimbursement entitlements in respect of medicines by Kela, the Social Insurance Institution of Finland - Repeated cross-sectional study	Apr 2019 – Mar 2022	Children (6–12 years)	≥ 1 prescription of an antidepressant within ATC N06A	Quarterly prevalence	n.r., but one external source identified [44]: approx. 423,164 6-12-year-olds (on December 31, 2022)	n.r.
Kuitunen, 2023 [36]	Finland	- Statistics on reimbursement entitlements in respect of medicines by Kela, the Social Insurance Institution of Finland - Repeated cross-sectional study	2019–2021	Adolescents and young adults (13–24 years)	≥ 1 prescription of an antidepressant within ATC N06A	1-year prevalence	n.r., but one external source identified [44]: approx. 732,531 13-24-year-olds (on December 31, 2021)	n.r.
Otter, 2024 [37]	Austria	- Routine data from the umbrella organization of Austrian social insurance institutions (The Federation of Social Insurances) - Repeated cross-sectional study	2013–2021	Individuals (age groups 10–14, 15–19 years, all age groups)	≥ 1 dispensation of an antidepressant within ATC N06A	Quarterly prevalence	n.r., but one external source identified [45]: approx. 423,433 10-14-year-olds (on January 1, 2021)	n.r.
Pedersen, 2024 [38]	Italy, Norway, Sweden	- National paediatric database Pedianet in Italy (covering 3% of the Italian paediatric population) and national prescription registers in Sweden (Swedish National Prescribed Drug Register) and Norway (Norwegian Prescription Database) - Repeated cross-sectional study	2018–2021	Children and adolescents (0–5, 6–11, 12–17 years, or 12–14 years for Italy)	≥ 1 prescription (Italy) or dispensation (Norway, Sweden) of an antidepressant within ATC N06A	Monthly prevalence	Total: 3,455,521 children and adolescents Italy: 136,188 Norway: 1,160,431 Sweden: 2,158,902 (in the year 2018)	n.r.; Total: 48.5%, Italy: 48.2%, Norway: 48.6%, Sweden: 48.5% (in the year 2018)
Valtuille, 2024 [39]	France	- X-ponent database from IQVIA dispensed prescriptions from 14,000 pharmacies (60% of all French pharmacies, without overseas territories) - Repeated cross-sectional study	Jan 2016 – May 2022	Children (6–11 years) and adolescents (12–17 years)	≥ 1 dispensation of an antidepressant within ATC N06A	Monthly prevalence	n.r. and no external source identified	n.r.

*ATC* Anatomical Therapeutic Chemical code, *NSMRIs* non-selective monoamine reuptake inhibitors, *SSRIs* selective serotonin reuptake inhibitors, *n.r.* not reported

* The prescription database for Norway only extended to 2020 and the Nordic Medico-Statistical Committees database (for all Nordic countries) only contains total estimates (not age-stratified). Therefore, Norway could not be included in our review

Of the eight studies, seven were repeated cross-sectional studies, and one was a retrospective cohort study. Weekly, quarterly or 1-year prevalences were reported by two studies each. One study reported weekly prevalences, while another reported utilization in terms of defined daily doses. All studies were based on secondary data (predominantly registry data) and defined antidepressant users as those who were prescribed at least one medication in the ATC (Anatomical Therapeutic Chemical) group N06A. Only two studies reported the exact number of young persons included. For five of the six studies, external sources were identified for providing the approximate number of young persons included the respective statistical database or registries. For Bojanić [33], two external sources were found from the national Danish [41] and Swedish registries [42]. For Garcia et al. [34], we found a source from the Spanish Statistical Office [43]. For both Finish studies [35, 36], numbers from Statistics Finland [44] were identified. For Otter et al. [37] one source was found from Statistics Austria [45]. For the French study Valtuille et al. [39] no external source could be identified, see Table 1.

### Quality assessment

The results of the quality assessment of all included articles are presented in detail in the supplementary file S2. An appropriate sample frame addressing the target population was identified in all eight studies. The requisite sample size, as defined by sample size calculations, was achieved in all eight studies. Two studies (25%) provided a detailed description of the study subjects and setting. All studies used valid methods in a standard, reliable way to identify antidepressant utilization, consistent with the nature of the administrative data on which they were all based. None of the studies reported an appropriate statistical analysis.

### Change of antidepressant utilization

Five studies reported overall findings on the change of antidepressant utilization among young persons [32, 33, 35, 36, 39]. Among these, two studies additionally reported results stratified by age [39] and by sex [32], respectively. One study reported results stratified by age only [38], one by sex only [34], and one by age and sex only [37]. Additionally, three of the eight studies reported not only the overall numbers for antidepressants but also values for individual agents or agent groups [32, 33, 36].

### Total prevalence

The data indicates a general increase in antidepressant utilization among European young persons during the course of the COVID-19 pandemic (see Table 2). The largest relative increase in overall antidepressant use was among French children aged 6-17-years [39], where monthly prevalence increased from 2.1 to 3.2 per 1,000 persons (+ 52%) between December 2019 and May 2022. Among Italian young adults aged 18–22 years [32], weekly prevalence increased from 9.2 to 13.3 per 1,000 persons (+ 44%) between the end of December 2019 and the end of October 2021. In Denmark [33], one-year prevalence among 5–19 years old children increased between 2018 and 2021 from 8.4 to 11.0 per 1,000 persons and in Sweden, from 19.7 to 24.3 per 1,000 persons. In Finland [35], quarterly prevalence among 6-12-year children increased from 1.7 to 2.1 per 1,000 persons (+ 24%) between the end of 2019 and the beginning of 2022, and annual prevalence among 13-24-year adolescents and young adults also rose from 65.9 to 82.3 per 1,000 persons (+ 25%) between 2019 and 2021 [36]. The studies for which results are only available stratified by age and sex also show an increase in the use of antidepressants (see Table 2).

**Table 2:** Results of the data included

Country (first author, year)^a^	Measure used in study and age range	Overall			By age/sex		
		Antidepressant utilization before the COVID-19 pandemic	Antidepressant utilization during the COVID-19 pandemic	Change	Antidepressant utilization before the COVID-19 pandemic	Antidepressant utilization during the COVID-19 pandemic	Change
Austria (Otter, 2024 [37])	- Quarterly prevalence per 1,000 persons − 10–19 years	n.r.	n.r.		**2019**,** 4th quarter**^**b**^: Total Females 10–14 years: 2.3 Females 15–19 years: 15.8 Males 10–14 years: 1.8 Males 15–19 years: 8.6	**2021**,** 4th quarter**^**b**^: Total Females 10–14 years: 4.2 Females 15–19 years: 23.0 Males 10–14 years: 2.0 Males 15–19 years: 9.4	+ 83% + 46% + 11% + 9%
Denmark ( Bojanić, 2024 [33])	− 1-year prevalence per 1,000 persons − 5–19 years	**2018**^**c**^: Total: 8.4 NSMRIs: 0.6 SSRIs: 7.1 Other antidepressants: 1.2	**2021**^**c**^: Total: 11.0 NSMRIs: 0.6 SSRIs: 9.5 Other antidepressants: 1.4	+ 31% ± 0% + 34% + 17%	n.r.	n.r.	
Finland ( Kuitunen, 2022 [35])	- Quarterly prevalence per 1,000 persons − 6–12 years	**2019**,** 4th quarter**^**c**^: Total: 1.7	**2022**,** 1 st quarter**^**c**^: Total: 2.1	+ 24%	n.r.	n.r.	
Finland ( Kuitunen, 2023 [36])	− 1-year prevalence per 1,000 persons − 13–24 years	**2019**: Total: 65.9 SSRIs: 48.0 Fluoxetine: 12.9 Citalopram: 3.3 Paroxetine: 0.9 Sertraline: 13.1 Escitalopram: 20.8 Other antidepressants: 27.2 Mirtazapine: 12.6 Bupropion: 4.2 Venlafaxine: 8.0 Duloxetine: 2.2 Vortioxetine: 4.0	**2021**: Total: 82.3 SSRIs: 60.8 Fluoxetine: 16.6 Citalopram: 2.7 Paroxetine: 1.1 Sertraline: 20.0 Escitalopram: 24.5 Other antidepressants: 34.0 Mirtazapine: 15.5 Bupropion: 5.9 Venlafaxine: 8.8 Duloxetine: 2.5 Vortioxetine: 6.6	+ 25% + 27% + 29% −18% + 22% + 53% + 18% + 25% + 23% + 40% + 10% + 14% + 65%	n.r.	n.r.	
France (Valtuille, 2024 [39])	- Monthly prevalence per 1,000 persons − 6–17 years	**Dec 2019**^**b**^: Total: 2.1	**May 2022**^**b**^: Total: 3.2	+ 52%	**Dec 2019**^**b**^: Total 6–11 years: 0.4 12–17 years: 3.8	**May 2022**^**b**^: Total 6–11 years: 0.4 12–17 years: 6.0	± 0% + 58%
Italy (Armando, 2024 [32])	- Weekly prevalence per 1,000 persons − 18–22 years	**Dec 23–29 2019**^**c**^: Total: 9.2	**Oct 25–31 2021**^**c**^: Total: 13.3	+ 44%	**Dec 23–29 2019**^**c**^: NSMRIs: females: 0.5 males: 0.8 SSRIs: females: 8.5 males: 6.3 Other antidepressants: females: 1.9 males: 1.4	**Oct 25–31 2021**^**c**^: NSMRIs: females: 0.7 males: 0.2 SSRIs: females: 13.6 males: 8.1 Other antidepressants: females: 2.7 males: 1.8	+ 34% + 100% + 60% + 29% + 44% + 29%
Italy (Pedersen, 2024 [38])	- Monthly prevalence per 1,000 persons − 0–14 years	n.r.	n.r.		**Dec 2019**^**b**^: Total 0–5 years: 0.0 6–11 years: 0.1 12–14 years: 0.2	**Dec 2021**^**b**^: Total 0–5 years: 0.0 6–11 years: 0.2 12–14 years: 0.6	± 0% + 100% + 200%
Norway (Pedersen, 2024 [38])	- Monthly prevalence per 1,000 persons − 0–17 years	n.r.	n.r.		**Dec 2019**^**b**^: Total 0–5 years: 0.0 6–11 years: 0.1 12–17 years: 2.6	**Dec 2021**^**b**^: Total 0–5 years: 0.0 6–11 years: 0.3 12–17 years: 4.7	± 0% + 200% + 81%
Spain (Garcia, 2023 [34])	- Daily Defined Doses for 1000 persons/day − 0–14 years	n.r.	n.r.		**2019**: Total females: 0.7 males: 0.6	**2021**: Total females: 1.2 males: 0.8	+ 71% + 33%
Sweden ( Bojanić, 2024 [33])	− 1-year prevalence per 1,000 persons − 5–19 years	**2018**^**c**^: Total: 19.7 NSMRIs: 0.5 SSRIs: 17.7 Other antidepressants: 3.4	**2021**^**c**^: Total: 24.3 NSMRIs: 0.7 SSRIs: 22.1 Other antidepressants: 3.9	+ 23% + 40% + 25% + 15%	n.r.	n.r.	
Sweden (Pedersen, 2024 [38])	- Monthly prevalence per 1,000 persons − 0–17 years	n.r.	n.r.		**Dec 2019**^**b**^: Total 0–5 years: 0.0 6–11 years: 1.0 12–17 years: 12.4	**Dec 2021**^**b**^: Total 0–5 years: 0.0 6–11 years: 1.2 12–17 years: 15.8	± 0% + 20% + 27%

*n.r.* not reported, *NSMRIs* non-selective monoamine reuptake inhibitors, *SSRIs* selective serotonin reuptake inhibitors,

^a^If a study is a multi-country study, it will be mentioned more than once

^b^Data for antidepressant utilization are shown in a figure in the article and corresponding data were extracted using WebPlotDigitizer by automeris

^c^Data for antidepressant utilization are shown in a figure in the article and exact data were provided on request from the authors

### Prevalence by age, sex and agents

The two studies that stratified results by age both found larger increases in older age groups. In France [39], prevalence increased by 58% in 12- 17-year-olds, whereas it remained stable in 6-11-year-olds. Pedersen et al. [38] examined monthly prevalences per 1,000 children and adolescents in three countries. The largest increases were found in the oldest age group: 12–14 years in Italy from 0.2 to 0.6 (+ 200%), 12–17 years in Norway from 2.6 to 4.7 (+ 81%) and 12–17 years in Sweden from 12.4 to 15.8 (+ 27%). In the lower age groups 0–5 and 6–11 years, the relative increases were high as well in all three countries, though the absolute prevalences were in a lower range (0.0 to 1.2 per 1,000 persons).

Two studies reported sex-stratified estimates only. In Spain [34], the defined daily doses for 1,000 females aged 0–14 increased from 0.7 to 1.2 (+ 71%) and for males of the same age from 0.6 to 0.8 (+ 33%). Armando et al. [32] reported results by sex only for three antidepressant subgroups. For non-selective monoamine reuptake inhibitors (NSMRIs), use increased more in males than in females (100% vs. 34%). For selective serotonin reuptake inhibitors (SSRIs) and other antidepressants, use increased more among females. The Austrian study by Otter et al. [37] showed that antidepressant use increased most among females in both age groups studied, 10–14 and 15–19 years. Between the fourth quarter of 2019 and the fourth quarter of 2021, use among 10-14-year-old females increased by 83% to 4.2 per 1,000 persons, while use among males in the same age group increased by 11% to 2.0 per 1,000 persons. Among females and males aged 15–19 years, prevalence increased by 46% and 9%, respectively.

In addition, data from three studies suggest that the use of SSRIs was more common than other classes of antidepressants (see Table 2). In Denmark [33], SSRIs constituted a considerable proportion of the observed increase in antidepressant utilization, with a 34% rise. The prevalence of SSRIs alone in 2021 was 9.5 per 1,000 persons (total antidepressants: 11.0). Other antidepressants also demonstrated an upward trend, albeit to a lesser extent. In Sweden [33], the relative increase of SSRIs was less pronounced than that of NSMRIs (25% vs. 40%), yet the prevalence of SSRIs was considerably higher than that of NSMRIs (22.1 vs. 0.7 per 1,000 persons). Similarly, in Finland [36], SSRIs exhibited notable increases, with sertraline rising by 53%. The prevalence of other antidepressants also increased by 25%. SSRIs were also the most common antidepressants in Italy. Armando et al. [32] also showed that their increase during the pandemic was mainly due to the increase in females (+ 60% vs. +29% in males).

## Discussion

During the COVID-19 pandemic, antidepressant utilization among European young persons increased considerably. Variations could be identified between countries, age groups and sexes, with notable increases of use in Italy and Scandinavia. Sex-specific trends showed higher increases in utilization among females, and SSRIs contributed markedly to the overall increase in antidepressant use.

The increasing use of antidepressants in European countries is likely driven by the prevalence of depression and anxiety disorders, which are commonly treated with these medications [46, 47]. Expanding their use to other psychiatric disorders (obsessive-compulsive disorders, insomnia, bipolar disorders, or anxious comorbidities in eating disorders, etc.) and physical conditions (e.g., peripheral neuropathic pain, migraine) has probably contributed to this increase. Our findings – and their public health implications – primarily relate to school-aged children, adolescents, and young adults. This reflects a concerning rise in mental health disorders especially within this demographic group. Most mental disorders manifest by age 14 years [48], however, the age at onset varies across different condition [49]. The COVID-19 pandemic has further exacerbated mental health challenges among youth. A meta-analysis reported that during the first year of the pandemic, approximately 1 in 4 youths globally experienced clinically elevated depression symptoms, and 1 in 5 experienced elevated anxiety symptoms [50]. In addition, a range of psychosocial and behavioral factors, including increased social media use and screen time, have also been identified as key contributors to deteriorating mental health among youth during the pandemic [51]. While these mechanisms were not explicitly addressed in the included studies, they provide important context for the rising demand for pharmacological treatment options in this population. Moreover, differences in the organization and accessibility of child and adolescent mental health services across European countries – as documented by Signorini et al. – may have influenced how emerging mental health needs were addressed, potentially affecting patterns of antidepressant prescribing [52].

While the relative changes in antidepressant utilization across countries were considerable, the absolute increases reported in most studies remained moderate in magnitude. The observed national differences are likely to reflect a combination of health system characteristics, cultural attitudes towards mental health and the unique impact of the pandemic and the respective restrictions in each country. Several of the included studies reported data from Nordic countries (Denmark, Sweden, Finland, and Norway [33, 35, 36, 38]), which allowed for a partial regional comparison. Increases in antidepressant utilization were observed across all included countries; however, the relative increases in the Nordic countries tended to be more moderate compared to those reported in other European settings. Nordic countries are known for spending more of their total healthcare expenditure on youth mental health services and a higher number of mental health personnel per head of population compared to other countries [53–55]. These structural advantages may have contributed to a higher level of resilience in these populations, potentially buffering the mental health impact of the pandemic and limiting increases in antidepressant use among young persons. Despite their advanced healthcare systems and high standard of living, the Nordic countries still face significant stigma toward people with mental illnesses. To address this issue, they have made efforts to raise public awareness of mental health disorders [56]. This could facilitate prompter diagnosis and treatment, potentially contributing to higher antidepressant use. However, the observed increases indicate that even well-resourced systems were not fully protected against rising mental health needs among youth. Further cross-country comparisons are needed to understand the role of system-level responses in moderating these trends. In Southern European countries, such as Italy, stigmas surrounding mental health and traditional family-centered approaches could lead to delays in seeking professional help [57]. Measures such as lockdowns, school closures and social distancing were likely to be associated with isolation and psychological distress [58]. For patients facing long waiting-lists for psychotherapy and limited access of in-person care and support services, the initiation or continuation of an antidepressant therapy could have been more practical for managing mental health symptoms than relying on a non-pharmacological approach. The intensity of the pandemic and related public health interventions varied widely between countries [59] which also explains the variability in trends of antidepressant utilization.

The COVID-19 pandemic has had a significant impact on mental health globally, leading to notable changes in antidepressant utilization among youth in various regions. In the USA, antidepressant prescriptions for adolescents and young adults increased by nearly 64% from 2020 to 2022 (December 2022: 4,284.8 per 100,000 persons). This trend was already escalating before the pandemic but accelerated during this period [60]. Similar results were found in a population-based study in Ontario with a relative change of + 21% between April 2020 and March 2022 [61]. In Australia, two studies provide further insight. Wood et al. [62] showed that the dispensing of antidepressants to children and adolescents doubled between 2013 and 2021, reaching 20.4 per 1,000 males and 33.8 per 1,000 females in 2021. A separate study by Hardie et al. [63] examined psychotropic prescribing by general practitioners during the pandemic and found that antidepressant prescribing for children and adolescents aged 5–19 years was significantly higher than expected, particularly among younger children. These findings suggest that while the level of antidepressant use varies internationally, the patterns observed in Europe during the pandemic are broadly consistent with trends in other high-income countries. Antidepressant utilization in the USA and Canada remains considerably higher than in Europe, whereas Australia shows similar levels. An earlier cross-national study by Bachmann et al. [64] also reported higher antidepressant use among young persons in the USA compared to several European countries.

The observed increases in antidepressant utilization should also be interpreted in the context of pre-existing trends. Some of the included studies provided data that extended several years prior to the pandemic, allowing for a more nuanced assessment of whether the changes observed during the pandemic reflect a continuation or intensification of earlier trajectories. Between 2006 and 2017, antidepressant use among 5–19-year-olds in Norway and Denmark [33] remained relatively stable, with prevalence fluctuating around 8–10 per 1,000 in Denmark and around 6–8 per 1,000 in Norway. In the study by Otter et al. [37], antidepressant prescribing among Austrian adolescents remained relatively stable between 2013 and 2019, especially in males, before rising more markedly during the pandemic years – particularly among females aged 10–19 years. Valtuille et al. [39] reported a steady increase in monthly antidepressant prescription among children and adolescents in France between 2016 and early 2020, followed by a more pronounced rise after the onset of the COVID-19 pandemic. Observed rates between 2020 and 2022 clearly exceeded the expected trend based on pre-pandemic patterns, suggesting a possible intensification of existing trajectories. These patterns suggest that the rise in antidepressant use among young persons was already underway in several countries prior to the pandemic, although the pandemic may have exacerbated the situation in some contexts.

The observed increase in antidepressant use among young females during the COVID-19 pandemic is consistent with well-documented gender differences in mental health. Female children and adolescents have high incidences in depressive, anxiety, eating, stress-related and bipolar disorders [65, 66] often leading to psychotropic drug therapy (e.g., with antidepressants). This has also been shown in large European studies for females of all ages [67, 68]. Increased antidepressant prescribing for female children and adolescents were found in the studies included in this review, as well as in the USA [60] and Australia [62]. This finding is consistent with research showing marked increases in suspected suicide attempts and hospitalizations and emergency department visits related to mental health among female adolescents after the pandemic outbreak [69, 70]. Taken together, this may indicate that the pandemic has exacerbated a pre-existing mental health crisis in this subpopulation.

SSRIs have become the predominant class driving the rise in antidepressant utilization among children and adolescents, as other studies looking at periods before the pandemic have also shown [71–73]. This trend reflects a preference for SSRIs in treating pediatric and adolescent mental health conditions, as it is consistent with established guidelines recommending SSRIs for depression and anxiety, e.g., in Sweden [74]. In line with the findings of this review, SSRIs, serotonin-norepinephrine reuptake inhibitors (SNRIs) and other newer antidepressants are being favored over older NSMRIs (tricyclic) globally [75]. However, NSMRIs are also used for other conditions such as pain, sleep disorders [76] and irritable bowel syndrome [77], which might contribute to their increasing use in Italy and Sweden as found in this review.

### Strengths and limitations

This is the first systematic review to summarise the change in antidepressant utilization in European children, adolescents and young adults over the course of the COVID-19 pandemic. The main strengths of the review are that a comprehensive search strategy was used, and no language restrictions were applied. We screened more than 4,400 titles and abstracts in three relevant databases. To further minimise the risk of missing studies we performed forward (citing) and backward (cited) citation analysis of the included studies using Web of Science Core Collection, which did not lead to the inclusion of additional studies. It is possible that we did not identify studies if they did not focus on the change of antidepressant utilization in young persons, but provided relevant information data in their full text. However, when screening the abstracts, we tended to check the full text instead to counteract this. In addition, the review also includes studies in which the results were presented in a graphical form only. There may also be studies that reported antidepressant use in young persons for different time points before and during the COVID-19 pandemic in separate publications. However, such studies would not have been included. Only two of the included studies reported their source and database populations, as recommended by the RECORD statement [78]; while this limited transparency was beyond our control, we attempted to compensate by estimating approximate population sizes using national data where possible. Although our eligibility criteria allowed both prevalence and incidence measures, only prevalence data were ultimately included, which enhanced the comparability and interpretability of results. As a consequence of our definition of the pre- and during-pandemic periods, the course of the year 2020 is not represented, although it may have marked a critical transitional phase. Another limitation is the heterogeneity in the length of time between the pre-pandemic and during-pandemic data points within each study, which ranged from 2 to 3.5 years. As longer observation periods may result in larger cumulative increases, this variability limits the comparability of the reported changes and may affect the interpretation of temporal trends. Relevant studies are probably still underway and will only be published in the near future. However, this is a highly relevant public health issue that requires synthesis of currently available data.

## Conclusion

The upward change in antidepressant utilization can be interpreted as part of a broader trend of increasing recognition and treatment of mental health issues in young populations. This surge may reflect the challenges faced by young persons during the COVID-19 pandemic. However, the variations in the extent of increase across countries and age groups also indicate differences in healthcare systems, diagnostic practices, and access to mental health care. The reasons for the increasing use, especially among females, should be investigated to determine if their benefits outweigh their harms, and if these medications were being used as substitutes for non-pharmacological therapy and support. Most of the studies included in this review analyzed data from Scandinavia, where health systems are more consistently oriented towards public health strategies and prevention. Nevertheless, there is still a lack of research on this issue, and longer-term trend analyses are needed to better understand underlying developments in mental health of young persons and antidepressant use beyond the context of the COVID-19 pandemic. Further analyses should also distinguish between different clinical indications for antidepressant prescribing to clarify whether increases reflect broader diagnostic trends, specific conditions such as depression, anxiety or pain, or shifts in treatment strategies.

## Supplementary Information

Below is the link to the electronic supplementary material.

Supplementary Material 1

Supplementary Material 2

Supplementary Material 3

## Data Availability

No datasets were generated or analysed during the current study.
