# Supplementary material for: Change of antidepressant utilization in children, adolescents and young adults in Europe before and during the COVID-19 pandemic: a systematic review
Source: Eur Child Adolesc Psychiatry. 2025 Aug 14;35(1):3–16. doi: 10.1007/s00787-025-02839-x (PMC12916912; doi:10.1007/s00787-025-02839-x)
Supplement: Supplementary file 1 — Supplementary Material 1 [file 787_2025_2839_MOESM1_ESM.docx]

Table S1. Search strategy

| **Sources for search** | **Search terms** |
| --- | --- |
| ***Database (platform)*** | |
| MEDLINE (PubMed)^a,b^ | ("antidepressive agents"[mh] OR "antidepressive agents, second generation"[mh] OR "serotonin and noradrenaline reuptake inhibitors"[mh] OR "neurotransmitter uptake inhibitors"[mh] OR "antidepressive agents, tricyclic"[mh] OR "monoamine oxidase inhibitors"[mh] OR "tryptophan"[mh] OR "antidepressant*"[tiab] OR "antidepressi*"[tiab] OR "psychotropic*"[tiab] OR "serotonin reuptake"[tiab:~2] OR "serotonin re-uptake"[tiab:~2] OR "serotonin uptake"[tiab:~2] OR "SSRI*"[tiab] OR "SNRI*"[tiab] OR "TCA"[tiab] OR "TCAs"[tiab] OR "tricyclic*"[tiab] OR "MAO inhibit*"[tiab] OR "monoamine oxidase inhibit*"[tiab] OR "RIMA*"[tiab] OR "MAOI*"[tiab] OR "agomelatin*"[tiab] OR "amitriptylin*"[tiab] OR "alaproclat*"[tiab] OR "amineptin*"[tiab] OR "amoxapin*"[tiab] OR "bupropion"[tiab] OR "butriptylin*"[tiab] OR "citalopram"[tiab] OR "clomipramin*"[tiab] OR "demexiptilin*"[tiab] OR "desvenlafaxin*"[tiab] OR "dibenzepin"[tiab] OR "dimetacrin*"[tiab] OR "duloxetin*"[tiab] OR "dosulepin*"[tiab] OR "dothiepin"[tiab] OR "doxepin"[tiab] OR "escitalopram"[tiab] OR "fluoxetin*"[tiab] OR "fluvoxamin*"[tiab] OR "imipramin*"[tiab] OR "iprindole"[tiab] OR "iproniazid*"[tiab] OR "isocarboxazid*"[tiab] OR "lofepramin*"[tiab] OR "levomilnacipran"[tiab] OR "maprotilin*"[tiab] OR "melitracen"[tiab] OR "metapramin*"[tiab] OR "milnacipran"[tiab] OR "minaprin*"[tiab] OR "mirtazapin*"[tiab] OR "moclobemid*"[tiab] OR "nialamid*"[tiab] OR "nortriptylin*"[tiab] OR "noxiptilin*"[tiab] OR "opipramol"[tiab] OR "paroxetin*"[tiab] OR "phenelzin*"[tiab] OR "pipofezin*"[tiab] OR "protriptylin*"[tiab] OR "quinupramin*"[tiab] OR "reboxetin*"[tiab] OR "sertralin*"[tiab] OR "tianeptin*"[tiab] OR "toloxaton*"[tiab] OR "tranylcypromin*"[tiab] OR "trimipramin*"[tiab] OR "venlafaxin*"[tiab] OR "vilazodon*"[tiab] OR "vortioxetin*"[tiab] OR "saint john*"[tiab] OR "tryptophan"[tiab]) AND ("Drug Prescriptions"[mh] OR "prescription*"[tiab] OR "prescrib*"[tiab] OR "dispens*"[tiab] OR "psychotropic use"[tiab:~1] OR "psychotropics use"[tiab:~1] OR "antidepressant use"[tiab:~1] OR "antidepressants use"[tiab:~1] OR "prevalence*"[tiab] OR "defined daily dose*"[tiab] OR "database"[tiab] OR "pharmacoepidemiolog*"[tiab] OR "pharmacotherap*"[tiab] OR (("prevalence"[tiab] OR "prescription"[tiab] OR "dispens*"[tiab]) AND ("rate"[tiab] OR "rates"[tiab]))) AND ("Infant"[mh] OR "Child"[mh] OR "Adolescent"[mh] OR "Minors"[mh] OR "Pediatrics"[mh] OR "Young Adult"[mh] OR "infant*"[tiab] OR "toddler*"[tiab] OR "child*"[tiab] OR "paediatric*"[tiab] OR "pediatric*"[tiab] OR "juvenil*"[tiab] OR "adolescen*"[tiab] OR "teen*"[tiab] OR "pupil*"[tiab] OR "student*"[tiab] OR "youth*"[tiab] OR "young"[tiab]) AND (2021/01/01:3000/12/31[dp]) |
| Emabse  (Ovid/Elsevier) ^a,b^ | ('antidepressant agent'/de OR 'serotonin noradrenalin reuptake inhibitor'/de OR 'serotonin uptake inhibitor'/de OR 'neurotransmitter uptake inhibitor'/de OR 'tricyclic antidepressant agent'/de OR 'monoamine oxidase inhibitor'/de OR 'tryptophan'/de OR 'antidepressant*':ti,ab OR 'antidepressi*':ti,ab OR 'psychotropic*':ti,ab OR 'ssri*':ti,ab OR 'snri*':ti,ab OR 'tca':ti,ab OR 'tcas':ti,ab OR 'tricyclic*':ti,ab OR 'mao inhibit*':ti,ab OR 'monoamine oxidase inhibit*':ti,ab OR 'rima*':ti,ab OR 'maoi*':ti,ab OR (serotonin NEXT/3 reuptake) OR (serotonin NEXT/3 're uptake') OR (serotonin NEXT/3 uptake) OR 'agomelatin*':ti,ab OR 'amitriptylin*':ti,ab OR 'alaproclat*':ti,ab OR 'amineptin*':ti,ab OR 'amoxapin*':ti,ab OR 'bupropion':ti,ab OR 'butriptylin*':ti,ab OR 'citalopram':ti,ab OR 'clomipramin*':ti,ab OR 'demexiptilin*':ti,ab OR 'desvenlafaxin*':ti,ab OR 'dibenzepin':ti,ab OR 'dimetacrin*':ti,ab OR 'duloxetin*':ti,ab OR 'dosulepin*':ti,ab OR 'dothiepin':ti,ab OR 'doxepin':ti,ab OR 'escitalopram':ti,ab OR 'fluoxetin*':ti,ab OR 'fluvoxamin*':ti,ab OR 'imipramin*':ti,ab OR 'iprindole':ti,ab OR 'iproniazid*':ti,ab OR 'isocarboxazid*':ti,ab OR 'lofepramin*':ti,ab OR 'levomilnacipran':ti,ab OR 'maprotilin*':ti,ab OR 'melitracen':ti,ab OR 'metapramin*':ti,ab OR 'milnacipran':ti,ab OR 'minaprin*':ti,ab OR 'mirtazapin*':ti,ab OR 'moclobemid*':ti,ab OR 'nialamid*':ti,ab OR 'nortriptylin*':ti,ab OR 'noxiptilin*':ti,ab OR 'opipramol':ti,ab OR 'paroxetin*':ti,ab OR 'phenelzin*':ti,ab OR 'pipofezin*':ti,ab OR 'protriptylin*':ti,ab OR 'quinupramin*':ti,ab OR 'reboxetin*':ti,ab OR 'sertralin*':ti,ab OR 'tianeptin*':ti,ab OR 'toloxaton*':ti,ab OR 'tranylcypromin*':ti,ab OR 'trimipramin*':ti,ab OR 'venlafaxin*':ti,ab OR 'vilazodon*':ti,ab OR 'vortioxetin*':ti,ab OR 'saint john*':ti,ab OR 'tryptophan':ti,ab) AND ('prescription'/exp OR 'prescription*':ti,ab OR 'prescrib*':ti,ab OR 'dispens*':ti,ab OR 'prevalence*':ti,ab OR 'defined daily dose*':ti,ab OR 'database':ti,ab OR 'pharmacoepidemiolog*':ti,ab OR 'pharmacotherap*':ti,ab OR (use NEAR/2 psychotropic*) OR (use NEAR/2 antidepressant*) OR (('prevalence':ti,ab OR 'prescription':ti,ab OR 'dispens*':ti,ab) AND ('rate':ti,ab OR 'rates':ti,ab))) AND ('infant'/exp OR 'child'/exp OR 'adolescent'/exp OR 'pediatrics'/exp OR 'young adult'/exp OR 'infant*':ti,ab OR 'toddler*':ti,ab OR 'child*':ti,ab OR 'paediatric*':ti,ab OR 'pediatric*':ti,ab OR 'juvenil*':ti,ab OR 'adolescen*':ti,ab OR 'teen*':ti,ab OR 'pupil*':ti,ab OR 'student*':ti,ab OR 'youth*':ti,ab OR 'young':ti,ab) AND  ([2021-01-01 to 3000-12-31]/pd) |
| PsycINFO (EBSCOhost) ^a,b^ | (DE "Antidepressant Drugs" OR DE "Serotonin Reuptake Inhibitors" OR DE "Serotonin Norepinephrine Reuptake Inhibitors" OR DE "Neurotransmitter Uptake Inhibitors" OR DE "Tricyclic Antidepressant Drugs" OR DE "Monoamine Oxidase Inhibitors" OR DE "Tryptophan" OR TI ("antidepressant*"OR "antidepressi*"OR "psychotropic*"OR "SSRI*"OR "SNRI*"OR "TCA" OR "TCAs" OR "tricyclic*" OR "MAO inhibit*" OR "monoamine oxidase inhibit*" OR "RIMA*" OR "MAOI*") OR AB ("antidepressant*"OR "antidepressi*"OR "psychotropic*"OR "SSRI*"OR "SNRI*"OR "TCA" OR "TCAs" OR "tricyclic*" OR "MAO inhibit*" OR "monoamine oxidase inhibit*" OR "RIMA*" OR "MAOI*") OR TI (serotonin W2 reuptake OR serotonin W2 re-uptake OR serotonin W2 uptake) OR AB (serotonin W2 reuptake OR serotonin W2 re-uptake OR serotonin W2 uptake) OR TI ("agomelatin*" OR "amitriptylin*" OR "alaproclat*" OR "amineptin*" OR "amoxapin*" OR "bupropion" OR "butriptylin*" OR "citalopram" OR "clomipramin*" OR "demexiptilin*" OR "desvenlafaxin*" OR "dibenzepin" OR "dimetacrin*" OR "duloxetin*" OR "dosulepin*" OR "dothiepin" OR "doxepin" OR "escitalopram" OR "fluoxetin*" OR "fluvoxamin*" OR "imipramin*" OR "iprindole" OR "iproniazid*" OR "isocarboxazid*" OR "lofepramin*" OR "levomilnacipran" OR "maprotilin*" OR "melitracen" OR "metapramin*" OR "milnacipran" OR "minaprin*" OR "mirtazapin*" OR "moclobemid*" OR "nialamid*" OR "nortriptylin*" OR "noxiptilin*" OR "opipramol" OR "paroxetin*" OR "phenelzin*" OR "pipofezin*" OR "protriptylin*" OR "quinupramin*" OR "reboxetin*" OR "sertralin*" OR "tianeptin*" OR "toloxaton*" OR "tranylcypromin*" OR "trimipramin*" OR "venlafaxin*" OR "vilazodon*" OR "vortioxetin*" OR "saint john*" OR "tryptophan") OR AB ("agomelatin*" OR "amitriptylin*" OR "alaproclat*" OR "amineptin*" OR "amoxapin*" OR "bupropion" OR "butriptylin*" OR "citalopram" OR "clomipramin*" OR "demexiptilin*" OR "desvenlafaxin*" OR "dibenzepin" OR "dimetacrin*" OR "duloxetin*" OR "dosulepin*" OR "dothiepin" OR "doxepin" OR "escitalopram" OR "fluoxetin*" OR "fluvoxamin*" OR "imipramin*" OR "iprindole" OR "iproniazid*" OR "isocarboxazid*" OR "lofepramin*" OR "levomilnacipran" OR "maprotilin*" OR "melitracen" OR "metapramin*" OR "milnacipran" OR "minaprin*" OR "mirtazapin*" OR "moclobemid*" OR "nialamid*" OR "nortriptylin*" OR "noxiptilin*" OR "opipramol" OR "paroxetin*" OR "phenelzin*" OR "pipofezin*" OR "protriptylin*" OR "quinupramin*" OR "reboxetin*" OR "sertralin*" OR "tianeptin*" OR "toloxaton*" OR "tranylcypromin*" OR "trimipramin*" OR "venlafaxin*" OR "vilazodon*" OR "vortioxetin*" OR "saint john*" OR "tryptophan")) AND (DE "Prescription Drugs" OR TI ("prescription*" OR "prescrib*" OR "dispens*" OR "prevalence*" OR "defined daily dose*" OR "database" OR "pharmacoepidemiolog*" OR "pharmacotherap*") OR AB ("prescription*" OR "prescrib*" OR "dispens*" OR "prevalence*" OR "defined daily dose*" OR "database" OR "pharmacoepidemiolog*" OR "pharmacotherap*") OR TI (use N1 psychotropic* OR use N1 antidepressant*) OR AB (use N1 psychotropic* OR use N1 antidepressant*) OR ((TI "prevalence" OR AB "prevalence" OR TI "prescription" OR AB "prescription" OR TI "dispens*" OR AB "dispens*") AND (TI "rate" OR AB "rate" OR TI "rates" OR AB "rates"))) AND (DE "Youth Mental Health" OR DE "Pediatrics" OR TI ("infant*" OR "toddler*" OR "child*" OR "paediatric*" OR "pediatric*" OR "juvenil*" OR "adolescen*" OR "teen*" OR "pupil*" OR "student*" OR "youth*" OR "young") OR AB ("infant*" OR "toddler*" OR "child*" OR "paediatric*" OR "pediatric*" OR "juvenil*" OR "adolescen*" OR "teen*" OR "pupil*" OR "student*" OR "youth*" OR "young")) AND (DT 20210101-30001231) |
| Notes:  ^a^Search strategies used in other systematic reviews for antidepressants (Zhou et al.^1^; Hetrick et al.^2^), drug prescriptions (Jobski et al.^3^) and Children/adolescents/young adults (Torbahn et al.^4^; Storebø et al.^5^) were adapted.  ^b^A search filter for identifying studies indexed in the databases between 1. January 2021 until the search date was adapted from a previous study (Hoffmann et al.^6^). | |

**References**

1. Zhou X, Teng T, Zhang Y, et al. Comparative efficacy and acceptability of antidepressants, psychotherapies, and their combination for acute treatment of children and adolescents with depressive disorder: a systematic review and network meta-analysis. *The Lancet Psychiatry*. 2020;7(7):581-601. doi:10.1016/S2215-0366(20)30137-1

2. Hetrick SE, McKenzie JE, Bailey AP, et al. New generation antidepressants for depression in children and adolescents: a network meta-analysis. *Cochrane Database Syst Rev*. 2021;2021(5). doi:10.1002/14651858.CD013674.pub2

3. Jobski K, Höfer J, Hoffmann F, Bachmann C. Use of psychotropic drugs in patients with autism spectrum disorders: a systematic review. *Acta Psychiatr Scand*. 2017;135(1):8-28. doi:10.1111/ACPS.12644

4. Torbahn G, Brauchmann J, Axon E, et al. Surgery for the treatment of obesity in children and adolescents. *Cochrane Database Syst Rev*. 2022;2022(9). doi:10.1002/14651858.CD011740.pub2

5. Storebø OJ, Storm MRO, Pereira Ribeiro J, et al. Methylphenidate for children and adolescents with attention deficit hyperactivity disorder (ADHD). *Cochrane Database Syst Rev*. 2023;2023(3). doi:10.1002/14651858.CD009885.pub3

6. Hoffmann F, Allers K, Rombey T, et al. Nearly 80 systematic reviews were published each day: Observational study on trends in epidemiology and reporting over the years 2000-2019. *J Clin Epidemiol*. 2021;138:1-11. doi:10.1016/j.jclinepi.2021.05.022
