# Supplementary material for: Change of antidepressant utilization in children, adolescents and young adults in Europe before and during the COVID-19 pandemic: a systematic review
Source: Eur Child Adolesc Psychiatry. 2025 Aug 14;35(1):3–16. doi: 10.1007/s00787-025-02839-x (PMC12916912; doi:10.1007/s00787-025-02839-x)
Supplement: Supplementary file 2 — Supplementary Material 2 [file 787_2025_2839_MOESM2_ESM.docx]

Table S2: Summary of quality assessment of all included articles

| **First Author, year** | **1** | **2** | **3** | **4** | **5** | **6** | **7** | **8** | **9** |
| --- | --- | --- | --- | --- | --- | --- | --- | --- | --- |
| Armando 2024 | Yes | Yes | Yes | Yes | Yes | Yes | Yes | No | N/A |
| Bojanić 2024 | Yes | Yes | Yes | No | Yes | Yes | Yes | No | N/A |
| Garcia 2023 | Yes | Yes | Yes | No | Yes | Yes | Yes | No | N/A |
| Kuitunen 2022 | Yes | Yes | Yes | No | Yes | Yes | Yes | No | N/A |
| Kuitunen 2023 | Yes | Yes | Yes | No | Yes | Yes | Yes | No | N/A |
| Otter 2024 | Yes | Yes | Yes | No | Yes | Yes | Yes | No | N/A |
| Pedersen 2024 | Yes | Yes | Yes | Yes | Yes | Yes | Yes | No | N/A |
| Valtuille 2024 | Yes | Yes | Yes | No | Yes | Yes | Yes | No | N/A |

*Abbreviations: N/A Not applicable*

Quality assessment criteria

| **1) Was the sample frame appropriate to address the target population?** | | |
| --- | --- | --- |
| Yes | Study focusing on the total population of children/adolescents/young adults |  |
| No | Study focusing on, for example, children from only one particular type of school or with a particular condition |  |
| Unclear | Unclear whether the sample included all children/adolescents/young adults or only a subgroup |  |
| N/A | - |  |
| **2) Were the study participants sampled in an appropriate way?** | |  |
| Yes | All children/adolescents/young adults considered or systematic sampled |  |
| No | Convenience sample |  |
| Unclear | Unclear whether all children/adolescents/young adults were considered or how the sampling was conducted |  |
| N/A | Administrative data |  |
| **3) Was the sample size adequate?** | |  |
| Yes | 384+ children/adolescents/young adults ((1.96^2*0.5*(1-0.5))/0.05^2) |  |
| No | <384 children/adolescents/young adults |  |
| Unclear | Number of included children/adolescents/young adults not reported and no indication that 384+ children/adolescents/young adults were included |  |
| N/A | - |  |
| **4) Were the study subjects and the setting described in detail?** | |  |
| Yes | Number of included children/adolescents/young adults, mean/median age of included children/adolescents/young adults, and proportion of females reported |  |
| No | Number of included children/adolescents/young adults, mean/median age of included children/adolescents/young adults, and proportion of females not reported |  |
| Unclear | - |  |
| N/A | - |  |
| **5) Was the data analysis conducted with sufficient coverage of the identified sample?** | |  |
| Yes | No children/adolescents/young adults with certain health conditions excluded and if consent to participate was required, it could be provided by proxies |  |
| No | children/adolescents/young adults with certain health conditions excluded or consent to participate could not be provided by proxies |  |
| Unclear | Unclear whether consent to participate could be provided by proxies |  |
| N/A | - |  |
| **6) Were valid methods used for the identification of the condition?** | |  |
| Yes | Antidepressant utilization information obtained based on records (includes, inter alia, questionnaires answered based on records), administrative data, or assessment |  |
| No | Information provided based on memory |  |
| Unclear | Unclear how the antidepressant utilization information was obtained or unclear whether records were reviewed to answer, for example, questionnaires |  |
| N/A | - |  |
| **7) Was the condition measured in a standard, reliable way for all participants?** | |  |
| Yes | Staff, health professionals, trained individuals, administrative data |  |
| No | Untrained individuals (with the exception of staff and health professionals) |  |
| Unclear | Unclear who obtained the antidepressant utilization information |  |
| N/A | - |  |
| **8) Was there appropriate statistical analysis?** | |  |
| Yes | Numerator and denominator regarding the antidepressant proportion reported |  |
| No | Numerator or denominator regarding the antidepressant proportion not reported |  |
| Unclear | - |  |
| N/A | - |  |
| **9) Was the response rate adequate, and if not, was the low response rate managed appropriately?** | |  |
| Yes | All children/adolescents/young considered, response >60%, or response <60% managed appropriately |  |
| No | Response <60% and no indication that it was managed appropriately |  |
| Unclear | Response not reported |  |
| N/A | Administrative data |  |
