# Supplementary material for: Change of antidepressant utilization in children, adolescents and young adults in Europe before and during the COVID-19 pandemic: a systematic review
Source: Eur Child Adolesc Psychiatry. 2025 Aug 14;35(1):3–16. doi: 10.1007/s00787-025-02839-x (PMC12916912; doi:10.1007/s00787-025-02839-x)
Supplement: Supplementary file 3 — Supplementary Material 3 [file 787_2025_2839_MOESM3_ESM.docx]

Table S3. Excluded records after full-text screening with reasons

| **Record** | **Reason for exclusion** |
| --- | --- |
| ***Database search*** | |
| Barker et al. Incidence of anxiety and depression in children and young people with life-limiting conditions. Pediatr Res. 2023 Jun;93(7):2081-2090. | 2 |
| Benefield et al. Patterns in School-Based Mental Health Visits and Psychotropic Medication Prescribing Before and During the COVID-19 Pandemic. School Mental Health (2024) 16:15–24 | 3 |
| Bliddal et al. Increasing use of antidepressants, hypnotics, and psychostimulants during the COVID-19 pandemic among Danish children, adolescents, and young adults. ABSTRACTS of ICPE 2022. No 197. | 1 |
| Bliddal et al. Psychotropic Medication Use and Psychiatric Disorders During the COVID-19 Pandemic Among Danish Children, Adolescents, and Young Adults. JAMA Psychiatry. 2023 Feb 1;80(2):176-180. | 5 |
| Boström et al. Antidepressant Use and Manic Episodes in Children and Adolescents With Unipolar Depression. JAMA Psychiatry. 2024 Apr 1;81(4):426. | 1 |
| Braillon and Rogers. Increased prescribing of psychotropic medication for children and adolescents during the COVID-19 pandemic: no cause for alarm. Med J Aust. 2023 Sep 18;219(6):285. | 1 |
| Bramness et al. The epidemiology of major depression among adults in Norway: an observational study on the concurrence between population surveys and registry data - a NCDNOR project. BMC Public Health. 2024 May 17;24(1):1330. | 2 |
| Carrasco-Garrido et al. Trends in the nonmedical misuse of benzodiazepines and Z-hypnotics among school-aged adolescents (2016-2021): gender differences and related factors. Child Adolesc Ment Health. 2024 Nov;29(4):345-354. | 2 |
| Estrela et al. Prescription of anxiolytics, sedatives, hypnotics and antidepressants in outpatient, universal care during the COVID-19 pandemic in Portugal: a nationwide, interrupted time-series approach. J Epidemiol Community Health. 2022 Apr;76(4):335-340. | 4 |
| Fuster-Casanovas et al. eHealth in the Management of Depressive Episodes in Catalonia's Primary Care From 2017 to 2022: Retrospective Observational Study. JMIR Ment Health. 2024 Jan 18:11:e52816. | 3 |
| Hardie et al. Psychotropic medication prescribing for children and adolescents by general practitioners during the COVID-19 pandemic. Med J Aust. 2023 Jul 3;219(1):26-27. | 7 |
| Harris. Antidepressant Prescriptions for Female Teens Surged After Pandemic. JAMA April 16, 2024 Volume 331, Number 15. | 1 |
| Hazell. Increased prescribing of psychotropic medication for children and adolescents during the COVID-19 pandemic: no cause for alarm. Med J Aust. Published online: 29 May 2023. | 1 |
| Hussey et al. Socio-demographic variation in diagnosis of and prescribing for common mental illnesses among children and young people during the COVID-19 pandemic: time series analysis of primary care electronic health records. J Child Psychol Psychiatry. 2025 Jan;66(1):16-29. | 3 |
| Keares et al. A Comparison of Pediatric Mental Health Diagnoses and Selective Serotonin Reuptake Inhibitor Prescribing Before and During the COVID-19 Pandemic. J Adolesc Health. 2023 Aug;73(2):387-389. | 3 |
| Kildegaard et al. Post-pandemic trends in psychotropic medication use in Danish children, adolescents, and young adults. Acta Psychiatr Scand. 2024 Sep;150(3):174-177 | 6 |
| Koball et al. The impact of adverse childhood experiences on healthcare utilization in children. Child Abuse Negl. 2021 Jan:111:104797. | 3 |
| Leong et al. Psychotropic Drug Use in Children and Adolescents Before and During the COVID-19 Pandemic. JAMA Pediatr. 2022 Mar 1;176(3):318-320. | 5 |
| Levaillant et al. Impact of COVID-19 pandemic and lockdowns on the consumption of anxiolytics, hypnotics and antidepressants according to age groups: a French nationwide study. Psychol Med. 2023 May;53(7):2861-2867. | 6 |
| Lien et al. Time trends in self-reported depressive symptoms, prescription of antidepressants, sedatives and hypnotics and the emergence of social media among Norwegian adolescents. PLoS One. 2023 Dec 27;18(12):e0295384. | 5 |
| McNicholas et al. Judicious Prescribing of Psychotropic Medication for Children and Adolescents. Ir Med J. 2022 Apr 29;115(4):576. | 1 |
| Nasir and Nasir. Antidepressant Prescriptions and Mental Health. Pediatrics. 2024 Mar 1;153(3):e2023064677. | 1 |
| Obermeier et al. From Recommendation to Implementation-Recommendations of the German Clinical Practice Guideline and Choice of Antidepressants for Children and Adolescents: Analysis of Data From the Barmer Health Insurance Fund. Dtsch Arztebl Int. 2021 Mar 26;118(12):215-216. | 3 |
| Pisarska et al. Prevalence and determinants of psychotropic medication use in Poland. Psychiatr Pol. 2024 Aug 31;58(4):619-636. | 4 |
| Robinson. Number of young children prescribed antidepressants has risen by 41% since 2015. The Pharmaceutical Journal, PJ, September 2021, Vol 307, No7953;307(7953). | 5 |
| Robinson. Peaks in number of young people prescribed antidepressants coincide with lockdowns. The Pharmaceutical Journal, PJ, June 2021, Vol 306, No7950;306(7950). | 6 |
| Robinson. A perfect storm: the impact of COVID-19 on the mental health of young people. The Pharmaceutical Journal, PJ, June 2021, Vol 306, No7950;306(7950): | 6 |
| Wallis et al. Lessons from the Netherlands for Australia: cross-country comparison of trends in antidepressant dispensing 2013-2021 and contextual factors influencing prescribing. Aust J Prim Health. 2024 Feb;30(1):NULL. | 5 |
| White et al. The Impact of COVID-19 on Psychotropic Medication Prescriptions in Adolescents: Analysis of a Federated Research Network. Adolescent Psychiatry. Volume 13, Issue 2, 2023, Published on: 05 October, 2023. | 5 |
| ***Forward and backward citation searching*** | |
| Aftab and Druss. Addressing the Mental Health Crisis in Youth-Sick Individuals or Sick Societies? JAMA Psychiatry. 2023 Sep 1;80(9):863-864. | 1 |
| Benton et al. Addressing the Global Crisis of Child and Adolescent Mental Health. JAMA Pediatr. 2021 Nov 1;175(11):1108-1110. | 1 |
| Creswell et al. Young people's mental health during the COVID-19 pandemic. Lancet Child Adolesc Health. 2021 Aug;5(8):535-537. | 1 |
| De Bandt et al. Prescriptions of antidepressants and anxiolytics in France 2012-2022 and changes with the COVID-19 pandemic: interrupted time series analysis. BMJ Ment Health. 2024 Feb 26;27(1):e301026. | 4 |
| Gonzalez-Lopez et al. Consumption of Psychiatric Drugs in Primary Care during the COVID-19 Pandemic. Int J Environ Res Public Health. 2022 Apr 14;19(8):4782. | 4 |
| Lamer et al. Prolonged increase in psychotropic drug use among young women following the COVID-19 pandemic: a French nationwide retrospective study. BMC Med. 2024 Jul 2;22(1):274. | 6 |
| Pieh. Assessment of Mental Health of High School Students During Social Distancing and Remote Schooling During the COVID-19 Pandemic in Austria. JAMA Netw Open. 2021 Jun 1;4(6):e2114866. | 2 |
| Vukicevic et al. Consumption of psychotropic drugs in Croatia before and during the COVID-19 pandemic: a 10-year longitudinal study (2012-2021). Soc Psychiatry Psychiatr Epidemiol. 2024 May;59(5):799-811. | 4 |
| Note: Reasons for exclusion:  1) Only congress abstract / other publication types (e.g. editorial, comment)  2) Antidepressant utilization not reported  3) No observational study / <100 persons per observation time point / specific subgroup  4) No children or young people up to 24 years  5) Not the required study years / change in utilization not reported  6) Not the required utilization measures  7) Studies from outside Europe  The assignment of the reasons for exclusion was done hierarchically in the order presented. |  |
